# An Elderly Woman With Gibbus Deformity and Physiologic Shock

**DOI:** 10.5811/cpcem.2019.6.42522

**Published:** 2019-08-14

**Authors:** Shuntaro Sakai, Yoji Hirayama, Motoji Oki

**Affiliations:** * Mutsu General Hospital, Department of Cardiology, Mutsu City, Aomori; † Hashimoto Municipal Hospital, Department of General Internal Medicine, Hashimoto, Wakayama; ‡ Hachinohe City Hospital, Department of Gastroenterology, Endoscope, and Chemotherapy, Hachinohe, Aomori

## Abstract

Physiological shock requires prompt diagnosis and treatment in the emergency department. We present a case of physiological shock in a 91-year-old woman resulting from obstruction of the left atrium and inferior vena cava by a giant esophageal hiatal hernia, identified using computed tomography imaging. The patient’s age and history, including diet and eating behavior (namely needing to lie down immediately after a meal), and kyphotic posture were important factors to consider in establishing the differential diagnosis. While rare, a giant esophageal hiatal hernia should be considered in the differential diagnosis of obstructive shock.

## CASE PRESENTATION

A 91-year-old Asian woman was admitted to our emergency department with complaints of nausea and dyspnea. One month prior to admission, the patient had become bedridden, and could maintain an upright sitting position for only 30 minutes. Moreover, she reported having to lie down quickly after meals. The patient, who was 143 centimeters tall and weighed 35 kilograms, had a significant thoracic gibbus deformity (extreme thoracic kyphosis from vertebral collapse and wedging).

Vital signs were as follows: a blood pressure of 89/52 millimeters of mercury, pulse of 123 beats per minutes, respiration of 32 breaths per minutes, and oxygen saturation (on ambient air) of 91%. Physical examination revealed epigastric tenderness, cool extremities, dry mouth, and livedo reticularis of both lower limbs. In addition to these physical findings, the laboratory evaluation was significant for a metabolic acidosis and hyperkalemia. There was no ST-T change on the electrocardiogram, and the transthoracic echocardiography was of poor quality, because of the patient’s short stature and kyphotic posture. We then performed chest radiography (CXR) and computed tomography (CT) (Image).

## DISCUSSION

The CXR showed a mass shadow on the mediastinum. CT showed the left atrium being compressed by a hiatal hernia. Obstructive shock, secondary to a giant esophageal hiatal hernia, was diagnosed. Both nasogastric tube and endoscopic examination failed to remove the stomach contents. The patient was not deemed to be a surgical candidate because of her advanced age and poor overall condition. After conservative treatment, she died with non-occlusive mesenteric ischemia on the following day.

The risk factors of esophageal hiatal hernia include female sex, aging, and gibbus.^1^ A hiatal hernia rarely leads to death. Only two previous similar cases have previously been reported in the literature and indicate that surgical management is necessary.^2^,^3^ We suggest that obstructive shock should be considered in elderly women, particularly in those with a gibbus deformity.

**CPC-EM Capsule**

What do we already know about this clinical entity?

*A hiatal hernia is well known as a common disease of aged people and it also causes reflux esophagitis*.

What is the major impact of the image(s)?

*Obstructive shock is a major type of shock, caused by pulmonary embolism, cardiogenic tamponade, and tension pneumothorax. However, a hiatal hernia can also cause obstructive shock*.

How might this improve emergency medicine practice?

*The obstructive shock caused by hiatal hernia should be considered in elderly women, particularly in those with a gibbus deformity*.

## Figures and Tables

**Image:**
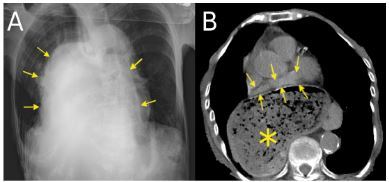
(A) Chest radiograph showing a mass shadow on the right side of the mediastinum (arrows). (B) Computed tomography of the chest in axial view showing displacement of the left atrium (arrows) by a giant esophageal hiatal hernia (stars).
